# Protecting Offspring Against Fire: Lessons From *Banksia* Seed Pods

**DOI:** 10.3389/fpls.2019.00283

**Published:** 2019-03-12

**Authors:** Jessica C. Huss, Peter Fratzl, John W. C. Dunlop, David J. Merritt, Ben P. Miller, Michaela Eder

**Affiliations:** ^1^Department of Biomaterials, Max Planck Institute of Colloids and Interfaces, Research Campus Golm, Potsdam, Germany; ^2^Department of Chemistry and Physics of Materials, University of Salzburg, Salzburg, Austria; ^3^Kings Park Science, Department of Biodiversity, Conservation and Attractions, Perth, WA, Australia; ^4^School of Biological Sciences, The University of Western Australia, Crawley, WA, Australia

**Keywords:** fire, seed protection, *Banksia*, follicle tissue, thermal insulation

## Abstract

Wildfires are a natural component in many terrestrial ecosystems and often play a crucial role in maintaining biodiversity, particularly in the fire-prone regions of Australia. A prime example of plants that are able to persist in these regions is the genus *Banksia*. Most *Banksia* species that occur in fire-prone regions produce woody seed pods (follicles), which open during or soon after fire to release seeds into the post-fire environment. For population persistence, many *Banksia* species depend on recruitment from these canopy-stored seeds. Therefore, it is critical that their seeds are protected from heat and rapid oxidation during fire. Here, we show how different species of *Banksia* protect their seeds inside follicles while simultaneously opening up when experiencing fire. The ability of the follicles to protect seeds from heat is demonstrated by intense 180 s experimental burns, in which the maximum temperatures near the seeds ranged from ∼75°C for *B. serrata* to ∼90°C for *B. prionotes* and ∼95°C for *B. candolleana*, contrasting with the mean surface temperature of ∼450°C. Many seeds of native Australian plants, including those of *Banksia*, are able to survive these temperatures. Structural analysis of individual follicles from these three *Banksia* species demonstrates that all of them rely on a multicomponent system, consisting of two valves, a porous separator and a thin layer of air surrounding the seeds. The particular geometric arrangement of these components determines the rate of heat transfer more than the tissue properties alone, revealing that a strong embedment into the central rachis can compensate for thin follicle valves. Furthermore, we highlight the role of the separator as an important thermal insulator. Our study suggests that the genus *Banksia* employs a variety of combinations in terms of follicle size, valve thickness, composition and geometric arrangement to effectively protect canopy-stored seeds during fire.

## Introduction

In many parts of the world, fire has been an integral force during the evolution of land plants (Pausas and Keeley, 2009). Plants possess a suite of traits, such as serotiny (Lamont, 1991; Lamont and Enright, 2000), smoke and heat-stimulated germination (Flematti et al., 2004; Pausas and Lamont, 2018), and post-fire resprouting and flowering (Pyke, 2017) to persist in fire-prone environments. All of these fire-related traits come along with temporal and structural adaptations of plant material, which prevent direct exposure of temperature-sensitive reproductive tissues to heat. Another strategy for fire survival is to rely on insulation and tissue protection provided by the soil, which may store seeds or protect resprouting rhizomes (Clarke et al., 2013).

Plants themselves can provide thermal insulation via increased resource investment into protective structures, such as thick bark (Pellegrini et al., 2017) or woody fruits (Lamont et al., 1991; Groom and Lamont, 1997). The storage of mature seeds inside woody fruits within the canopy (i.e., serotiny) and their release after fire provides fitness benefits to plants in fire-prone regions (Causley et al., 2016). Despite the abundance of plants with serotinous fruits in frequently burnt regions (Groom and Lamont, 2015), the material properties of tissues that enable thermal insulation during fire remain largely unknown. Studies on tree bark show that thermal insulation during fire depends on the moisture content, density and thickness of the protective layer (Bauer et al., 2010; Lawes et al., 2011; Pellegrini et al., 2017). Similarly, Bradstock et al. (1994) found that for the canopy-held fruits of different species of *Hakea*, fruit size – in particular the thickness of the fruit walls (follicles) – is crucial for seed survival during fire. Regardless of the type of seed storage (canopy or soil), Hanley and Lamont (2000) found that seed survival generally depends on the exposure time and temperature; highlighting the importance of thermal insulation for seed survival. From Fourier’s law of heat conduction, it is known that not only does the thickness of a material play a role, but so does its surface area and conductivity, in addition to the external and internal temperature, which change as a function of time during fire. Therefore, thickness alone might not be sufficient to indicate how well fruits insulate seeds in fire-prone ecosystems. Despite the efforts that have been made to determine the lethal temperatures of seeds inside fruits (Judd, 1993; Bradstock et al., 1994; Habrouk et al., 1999; Hanley and Lamont, 2000), the fruit tissue itself remains largely unstudied.

**FIGURE 1:**
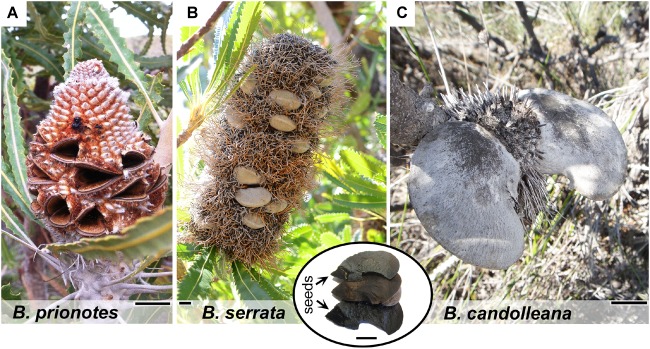
Morphology of mature cones and follicles of different *Banksia* species. **(A)** Fruit of *B. prionotes* with open follicles and seeds already released. **(B)** Mature infructescence of *B. serrata* on the plant showing closed follicles surrounded by dried, flammable florets (photo by S. Ehrig, reproduced with permission). Inset: content of a single follicle with position of the seeds indicated. **(C)** Typical fruit of *B. candolleana* with large follicles. All scale bars: 1 cm.

In the following sections, we address these issues by exploring the insulating properties of the follicles in different species of *Banksia*, native to fire-prone regions in Australia. The three species investigated – *B. prionotes*, *B. serrata* and *B. candolleana* – differ not only in their distribution and ecology, but also in the morphology of their fruits (infructescences). Besides the different arrangement of follicles on the central rachis, the follicles vary in size and geometry (Figure 1A–C). Each follicle usually contains 1–2 seeds that are stored in the back part; at the follicle base and furthest away from the opening. A thin wing is attached to the seed on its exterior side (Figure 1B inset), which assists its dispersal with the wind. The selected species for this study differ in their response to fire: *B. prionotes* plants are killed by fire and rely entirely on seeds for regeneration, whereas *B. serrata* and *B. candolleana* are able to survive fire and regrow from epicormic and lignotuberous buds after the passage of fire, as well as regenerate from seeds (Collins et al., 2008). A large number of *Banksia* species, including *B. prionotes* and *B. candolleana*, occur in the biodiverse southwest of Western Australia (Taylor and Hopper, 1988), where their fruits face variable exposure to fire depending on climate, vegetation structure, fuels and fire regime. In contrast, *B. serrata* occurs along the east coast of Australia, where different climatic conditions and fire regimes are dominating (Whelan et al., 1998; Collins et al., 2008). Studies conducted in bushland have indicated that the average flame front residence time, with *T* > 200°C during experimental fire, lies between *t* = 25–125 s in a *Banksia* shrubland (Enright and Lamont, 1989). Similar results (*t* = 37 s) are given by Wotton et al. (2012) for a eucalypt forest with maximum flame temperatures recorded in the range of *T* = 300–1100°C. Here, we study the follicles of three *Banksia* species to understand which structural and thermal properties govern seed protection during fire – while the follicles are opening.

## Results

### Temperatures During Experimental Burns

**FIGURE 2:**
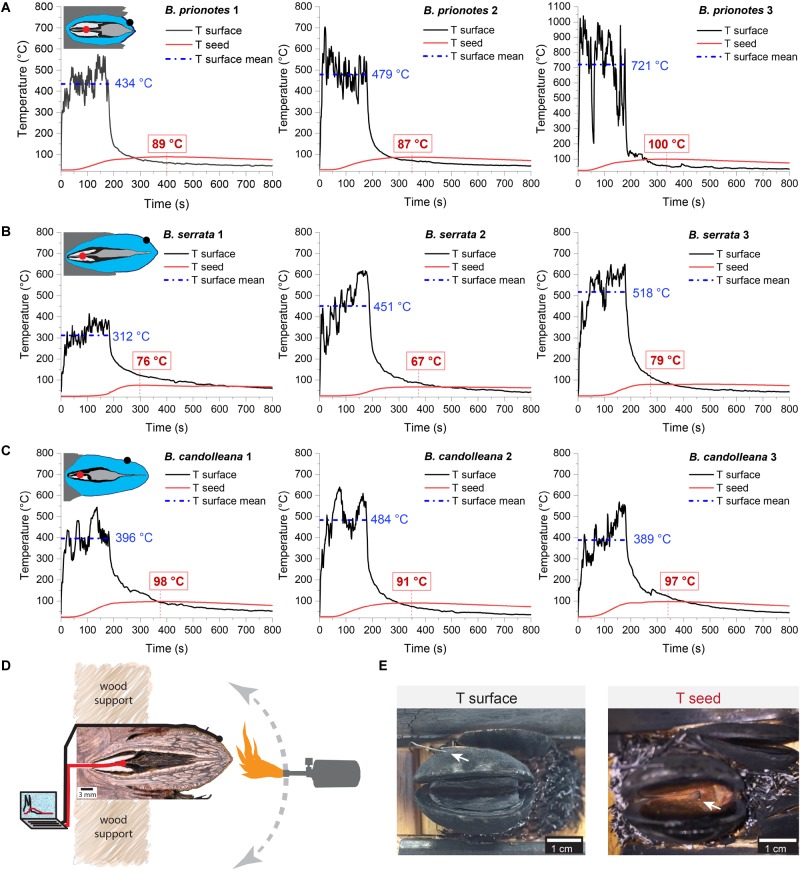
Experimental burns (180 s) of follicles with different geometries. Temperatures were recorded on the surface of the upper valve (means during burning indicated in blue) and inside the follicle near the seed (maximum indicated in red) for three follicles of **(A)** *B. serrata*, **(B)** *B. candolleana* and **(C)** *B. prionotes*. Icons are schematic drawings based on longitudinal sections of follicles, dots show the approximate position of the two thermocouples (*T_surface_* in black and *T_seed_* in red). **(D)** Experimental set-up for the 180 s burns: before burning, the mechanically isolated follicles were glued onto wood support holders at their embedment (the image shows a longitudinally sectioned follicle to illustrate the position of the thermocouples). The positions of the two thermocouples are indicated by the black and red ‘wires’. During burning, a Bunsen burner was manually rotated around the follicle for 180 s while recording the temperatures on the surface and near the seed(s). **(E)** Front views of follicles after burning. (*left*) the outer thermocouple was always positioned on the surface of the upper valve (arrow), measuring *T_surface_*. (*right*) The thermocouple near the seed, measuring *T_seed_*, was inserted through a small drill hole at the follicle base and is only visible after burning and removal of the separator and the seeds. Scratches on the seeds often confirmed that the thermocouple was placed centrally and directly on top of or below a seed.

**FIGURE 3:**
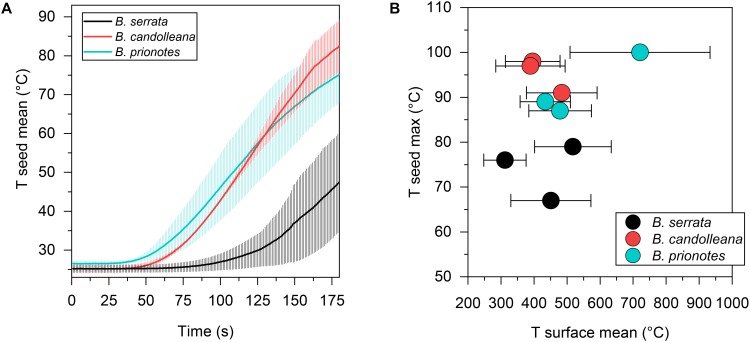
Seed temperatures during the experimental burns. **(A)** Mean seed temperature (±SD, *n* = 3) for all three species as a function of time, revealing a slow temperature response in *B. serrata* and a quick response in *B. prionotes* during burning of follicles for 180 s. **(B)** Maximum seed temperatures plotted against the mean surface temperature (±SD) for each burn shown in Figure 2. *B. serrata* shows the lowest maximum seed temperature when compared to the other two species for a similar mean surface temperature.

In our study, we tested the fire tolerance of single follicles by exposing them to the flame of a rotating Bunsen burner for *t* = 180 s, which mimics an intense fire event. During these experimental burns, the follicle surface did not ignite, indicating that the production of flammable volatiles did not occur in a sufficient ratio relative to oxygen to maintain a flame. Over the 180 s of burning, mean temperatures at the follicle surface (*T_surface,mean_*) ranged from 312°C to 721°C (Figure 2A–C). Large fluctuations of *T_surface_* may arise from rotating the flame during the experiment, which can lead to short discontinuities of flame exposure (Figure 2D,E). For all three species, there was a delayed increase in the temperature inside the follicles near the seeds (*T_seed_*), compared to surface temperatures, with the maximum (*T_seed,max_*) occurring after the end of the burn at *t* > 180 s (Figure 2A–C). The data obtained from the experimental burns were analysed by comparing the three species with regards to the temperature profiles during burning (Figure 3A), and *T_seed,max_* of each burn in relation to *T_surface,mean_* (Figure 3B). The average time lag before any increment in *T_seed_* occurs is longer in *B. serrata* follicles, compared to *B. prionotes* and *B. candolleana* (Figure 3A). Interestingly, *T_seed,max_* seems to be only slightly affected by the external temperature, because similar values, within and between species, are reached for *T_seed,max_* despite strong variations of *T_surface,mean_* between different burns (Figure 3B).

### Structural and Thermal Properties of the Follicles

**FIGURE 4:**
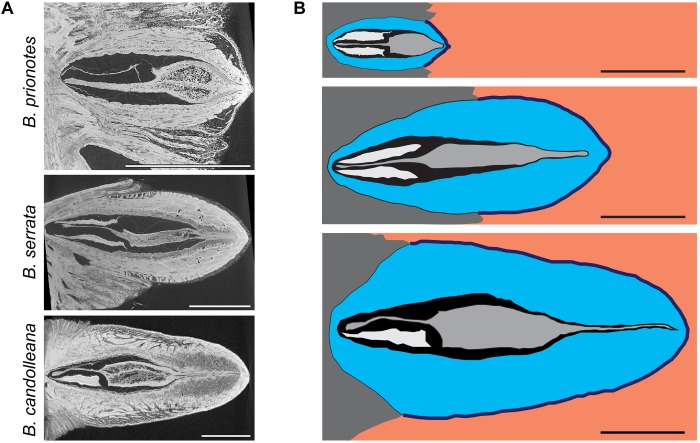
Structural and geometrical analysis of follicles. **(A)** Micro-CT images of follicles in the longitudinal view: *B. prionotes* follicles show a porous exocarp in contrast to the other two species (this follicle aborted both seeds). A highly porous separator can be identified in all three species. **(B)** Schematic drawing based on longitudinal sections of follicles from all three species (top to bottom: *B. prionotes, B. serrata, B. candolleana*) showing the differences in size, geometry and embedment of all follicles and their components (valves in blue, seed separator in grey, seeds in white/ wings not displayed, embedment in dark grey). Orange area indicates areas with direct exposure to flames. Sometimes only one (as seen in *B. candolleana* here) or no seed is developed. All scale bars: 1 cm.

Before discussing the variation among *T_seed,max_* values obtained for the three species, the respective tissue properties and follicle geometry need to be considered. In the longitudinal view of the X-ray microtomography (CT) images (Figure 4A), pronounced differences in tissue structure and density can be identified: follicles of *B. prionotes* have thin valves with a porous outermost layer (exocarp), whereas those of *B. serrata* and *B. candolleana* have thicker valves, lacking porosity in the exocarp. In all species, the seeds and follicle components are arranged in a distinct manner, which is crucial to survive fire: a woody separator holds the seeds in place within the follicles by tightly packing the wing of each seed in between a follicle valve and the separator in the tip region, where the follicle opens (Figure 4A). Furthermore, each seed is surrounded by a thin layer of air with a thickness of ∼0.5 – 1 mm. All separators show a thickened and highly porous region in their centre, which is likely to limit heat transfer toward the seeds due to its porous structure. The geometry of this porous region of the separator varies between species: the centre is thinnest in *B. serrata*, and it is located closer to the valve tip in *B. prionotes*. It is noteworthy that this thickened part of the separator forms the main barrier between the seeds and the exterior once the follicles are open (after ∼20 – 30 s of flame exposure), because of a simultaneous deformation of the paired wing-like tip of the separator (Figure 1B inset), which opens together with the valves. The schematic drawings (Figure 4B) highlight the differences in size and geometry, and reveal a varying embedment of follicles into the rachis for the three species: *B. prionotes* has strongly embedded follicles, whereas *B. serrata* follicles are about 50% embedded. The large follicles of *B. candolleana* show the least embedment into the rachis at the follicle base. Therefore, the exposed follicle area is largest for *B. candolleana*, followed by *B. serrata* and it is smallest in *B. prionotes*.

**FIGURE 5:**
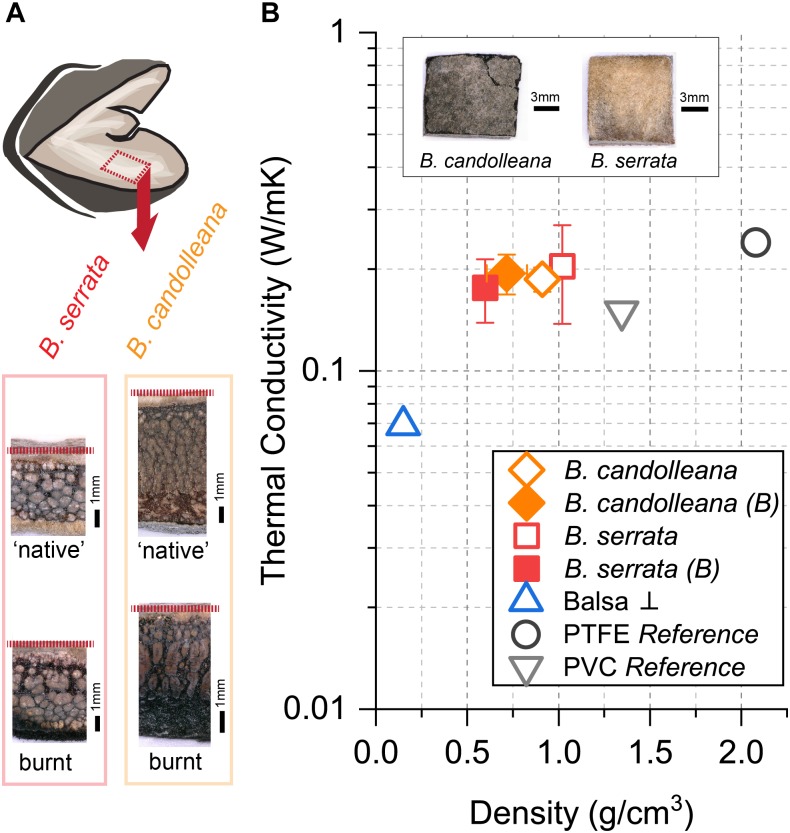
Thermal conductivity of follicle valves in comparison to reference materials. **(A)** Position of samples and transverse view of ‘native’ (non-burnt) and burnt samples, showing the layered tissue structure and sample thickness (dashed red line indicates the location of the endocarp/innermost valve tissue). **(B)** Thermal conductivity at ∼43°C for non-burnt and burnt *(B)* valve samples of *B. candolleana* (mean ±SD, *n* = 3) and *B. serrata* (mean ±SD, *n* = 3) compared to other materials as a function of density. The outer surfaces of exemplary valve samples are shown on the images (non-burnt). Moisture content *MC* ≈ 8% for non-burnt follicles, flame exposure of the follicle surface for *t* = 30 s for burnt samples. Values of PTFE and PVC are taken from reference and samples were used for set-up calibration. Commercial Balsa wood (*MC* ≈ 5%, *n* = 1) was measured perpendicular ⊥ to the grain and used as a standard to test the accuracy of our simple set-up.

During wildfires, the first follicle components that face heat, or fire directly, are the valves. Therefore, we experimentally determined the thermal conductivity *k* of samples from the valve tissue (Figure 5A) of *B. candolleana* and *B. serrata.* By recording the temperature gradient between the heated follicle surface and the inside of the valve (Figure 5A), we found in both species that *k* ≈0.2 W/mK (Figure 5B) for non-burnt samples, and also for burnt samples with a 1–2 mm thick char layer on the surface. No experimental results are available for the thermal conductivity of valves in *B. prionotes.* However, we expect *k* to be lower for *B. prionotes* due to the presence of a porous exocarp, which leads to a reduced density, and thermal conductivity, if we assume that these two parameters are linearly correlated, as shown for wood and bark (Hengst and Dawson, 1994; Suleiman et al., 1999).

### Heat Transfer Through the Valves

**FIGURE 6:**
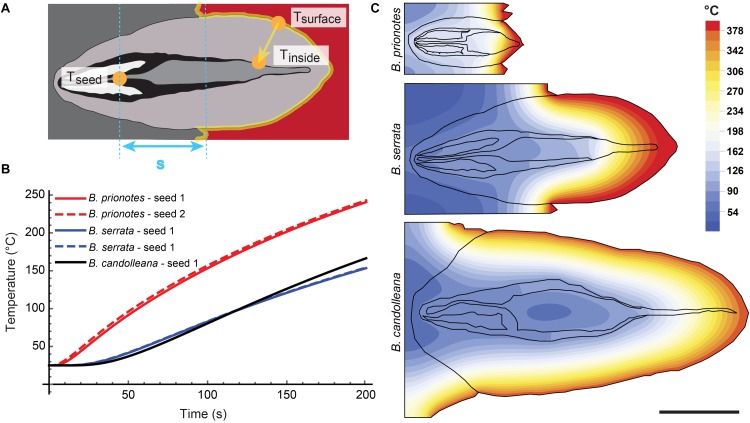
Simulation of heat transfer in closed follicles. **(A)** Schematic drawing showing the relevant locations for temperature measurements and calculations in a closed follicle. **(B)** Seed temperatures as a function of time for the three species, obtained from simulations with initial temperature gradients similar to the experimental burns: Δ*T* (*B. prionotes*) = 414°C (Figure 2A1); Δ*T (B. serrata)* = 431°C (Figure 2B2); Δ*T* (*B. candolleana*) = 376°C (Figure 2C1). **(C)** 2D simulations for all three species showing the local temperatures at *t* = 180 s with the same parameters used as in B. In the simulation, the follicles remained closed and different material parameters were only assigned to air; all solid components have the same properties (valves, separator, seeds, embedment) to reveal the role of geometry. Scale bar: 1 cm

The follicles exhibit multiple anisotropic tissue layers: each valve consists of at least three distinct layers, namely exo-, meso-, and endocarp, followed by a layer of air and another layer formed by the separator (see Figure 4A). Furthermore, the valves open while being heated. During the initial phase of heating, as schematically illustrated in Figure 6A, heat primarily diffuses through the valves of thickness *x*. The thermal diffusivity α depends on *k*, but also on the tissue density ϱ and specific heat *c* (eq. 1). A first approximation for the heat transfer through the valves can be obtained by calculating the time that is required for *T_inside_* to increase by half of the initial Δ*T* between *T_surface_* and *T_inside_* (expressed as Δ*t*_50_, eq. 1,2, Carslaw and Jaeger, 1959).

(1) $$\alpha =\frac{k}{\varrho c}$$

(2) $$\Delta t_{50}\approx \frac{x^{2}}{\alpha }$$

An example for this would be the time that is required to obtain 190°C for *T_inside_*, given that *T_surface_* is constantly kept at 400°C (with an initial *T_inside_* = 20°C; thus an initial Δ*T* = 380°C). The approximation yields Δ*t*_50_ = 34 s for *B. prionotes*, Δ*t*_50_ = 242 s for *B. serrata* and Δ*t*_50_ = 347 s for *B. candolleana* (Table 1). This shows that heat is being transferred quickly through *B. prionotes* valves, followed by *B. serrata* and it is transferred slowest for *B. candolleana*, in which heat needs about 10 times longer than in *B. prionotes* to diffuse through the valves (Table 1). As a consequence of this, *B. candolleana* follicles might also open up later than those of *B. prionotes*, because a critical temperature is required in the endocarp to initiate follicle opening (Huss et al., 2018).

**Table 1:** Overview of different follicle parameters.

Parameter	*B. prionotes*	*B. serrata*	*B. candolleana*
*k* (W/mK)	0.10^∗^	0.20	0.19
*x* (m)	0.002	0.006	0.007
*A* (m^2^)	2.44E-04	1.43E-03	2.16E-03
*ϱ* (kg/m^3^)	500^∗^	800	800
Δ*t*_50_ *(s)*	34^†^	242^†^	347^†^
*Avg_T*_*seed*,180*s*_ *(°C)*	75.1 ± 7.2	47.5 ± 12.7	82.5 ± 6.8
*c* (J/kgK)	1680^∗^	1680^∗^	1680^∗^
α (m^2^/s)	1.19E-07^†^	1.49E-07^†^	1.41E-07^†^
*T_surface,mean_ -T_inside_* (°C)	414 (*A1*)	431 (*B2*)	376 (*C1*)
*Exp_T*_*seed*,180*s*_ (°C)	71 (*A1*)	45 (*B2*)	85 (*C1*)
*Th_T*_*seed*,180*s*_ (°C) (closed follicles)	226^†^	141^†^	151^†^

*k: thermal conductivity of the valves; x: valve thickness; A: follicle surface area, corresponding to ½ A of a triaxial ellipsoid with similar dimensions as the follicles; **ϱ**: valve density; Δt_50_: time required to reach half of the temperature difference between T_surface_ and T_inside_; Avg_T_seed,180s_: average temperatures (±SD, n = 3) near the seed at t = 180 s; c: heat capacity; α: thermal diffusivity; T_surface,mean_ - T_inside_: initial temperature difference (based on individual measurements; references indicate measurements in Figure 2); Exp_T_seed,180s_: measured temperatures near the seed at t = 180 s; Th_T_seed,180s_: calculated temperatures near the seed at t = 180 s. ^∗^Estimated values based on similar reference materials. ^†^Theoretical results from calculations.*

To estimate the role of geometry on heat transfer, we discretized and meshed the 2D images of longitudinal sections of follicles from all species (Figure 4B). The heat equation was then solved on these meshes with the Finite Element (FE) method (Figure 6B,C) using the initial experimental temperature gradients: Δ*T (B. prionotes)* = 414°C (Figure 2A1); Δ*T (B. serrata)* = 431°C (Figure 2B2); Δ*T (B. candolleana)* = 376°C (Figure 2C1). *B. prionotes* was predicted to heat up more quickly, with little difference in the heating rates between *B. serrata* and *B. candolleana* (Figure 6B). The absolute values of the seed temperatures achieved after 180 s are much higher than the measured temperatures, (FE Simulation: ∼226°C for *B. prionotes*; ∼141°C for *B. serrata* and ∼151°C for *B. candolleana* vs. experimental data: 71°C for *B. prionotes*; 45°C for *B. serrata*; 85°C for *B. candolleana*). It is possible that this is due to the simplified boundary conditions that do not consider any change in thermal conductivity or composition due to charring or to opening of the follicle during heating.

### A Network of Antioxidative Tissue

**FIGURE 7:**
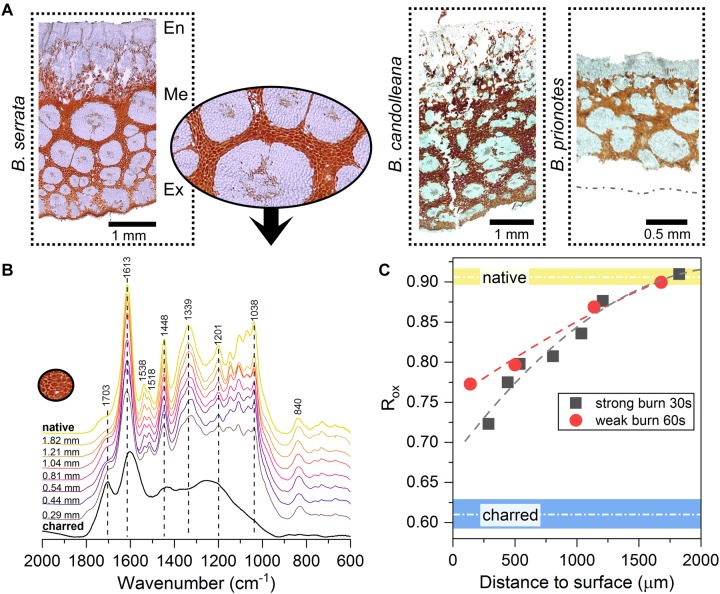
Characterisation of the anti-oxidative tannin network in the valve tissue. **(A)** Light micrographs of unstained follicle cross-sections from all three *Banksia* species, showing networks of tannins-rich parenchymatic tissue (in red/orange, inset for *B. serrata*) surrounding thick-walled fibre bundles (white) in the mesocarp (Me). En: endocarp; Ex: exocarp. **(B)** FT-IR spectra of *B. serrata* tannins from different depths below the surface of a burnt follicle (30 s of flame exposure, distance to the follicle surface indicated) compared to ‘native’ and charred. **(C)** Ratio of aromatic to carboxylic absorption bands [1613 cm^−1^/(1703 cm^−1^ ++ 1613 cm^−1^)] for samples with two different flame exposures allows to quantify the state of the condensed tannins. Short burnings of 30 and 60 s mainly affect the tissue in the outermost valve layer and causes oxidation of the condensed tannins to a depth of only 1.5–2 mm below the surface. Data fitted with a 2nd order polynomial function, native and charred are averages obtained from 4 spectra ± SD.

As revealed by Fourier-Transform Infrared (FT-IR) spectroscopy and light microscopy, all three species contain condensed tannins in the parenchymatic tissue of the valves (Figure 7A). However, *B. serrata* seems to contain the largest amounts among the three species. Therefore, a detailed analysis of these compounds was performed for *B. serrata*, showing that the tannins are mainly comprised of (epi-) gallocatechin units, indicated by the clear double peak in the spectral region from 1510 to 1550 cm^−1^ in Figure 7B, which arises from vibrations of the hydroxyl ring substituents (Ricci et al., 2015). The same compounds were previously reported for the species *B. attenuata* (Huss et al., 2018). During fire, all components that are facing flames are subject to rapid thermal and chemical degradation via bond dissociation, radical formation and oxidation within a short period of time. Heat exposure of the condensed tannins results in molecular modifications: with increasing exposure (closer to the surface) the bands of hydroxyl groups are seen to decrease (1201 cm^−1^ and 1538 cm^−1^) and carbonyl bands are seen to increase (*C* = O stretching band at 1703 cm^−1^). The spectrum of the charred samples show similar bands as charred lignin after a treatment of 8 h at 400°C (Rutherford et al., 2005).

By calculating the intensity ratio of absorption bands of aromatic carbons relative to carbonyl groups (inspired by Kister et al., 1990), according to eq. 3, the oxidation can be traced within the tannin-enriched parenchymatic tissue before and after burning to quantify the effect of flame exposure (Figure 7C).

(3) Rox=I1613 cm−1I1703 cm−1+I1613 cm−1

As expected, the parenchymatic tissue shows a pronounced oxidation gradient from the out- to the inside. The tannins right below the surface undergo stronger oxidation during burning, whereas the tannins closer to the endocarp (>1.5–2 mm below the surface; Figures 7B,C), remain unchanged when compared to the native state. Interestingly, despite being close to the surface, the tannins measured at a depth of only 0.29 mm still differ from the charred samples (Figure 7B). On the one hand, this can be explained by the presence of a char layer on the surface, which can act as a barrier for gases and heat, thereby slowing down thermal decomposition (Lowden and Hull, 2013). On the other hand, this could also be a direct effect of the hydroxyl-rich moieties of the tannins, which are known to act as effective antioxidants.

## Discussion

In *Banksia* woodlands and shrublands, crown fires occur at average intervals of 15–25 years (Bond and van Wilgen, 1996), but may also be observed earlier or later in different regions (Enright et al., 2012). Experimental burns in *Banksia* shrublands demonstrate that during crown fires, a fruit is usually exposed to fire for a maximum time of 60–120 s (Enright and Lamont, 1989). We chose an exposure of *t* = 180 s to simulate an intense burning event. Therefore, the temperatures that are typically reached near the seeds during a wildfire are likely to be lower than our reported maximum temperatures.

Our findings demonstrate that the follicles of all three study species sufficiently insulate seeds to a maximum temperature of c. 70–100°C over *t* = 180 s of heating, even when maximum surface temperatures commonly exceed 500 – 600°C, and reach as high as 1000°C in *B. prionotes*. Despite great differences in follicle size, geometry, arrangement and structure among species, they all seem to provide sufficient insulation during fire. In *B. prionotes*, follicles are small, strongly embedded and rely on thin-walled valves with a highly porous exocarp. These properties result in a low thermal diffusivity through the valves and a small surface area exposed to heat. In contrast, *B. candolleana* is characterised by large, thick-walled and almost free standing follicles, in which the thick valves play a key role for insulation due to the lack of embedment. The medium sized follicles of *B. serrata* show the largest distance between the seeds and closest surface exposed to heat (distance *s*, Figure 6) and a large proportion of tannin-enriched parenchymatic tissue in the valves (Figure 7).

Despite the strong differences in heat transfer rates between *B. prionotes* and *B. candolleana*, the follicle arrangements of both species perform equally well in the end, because similar temperatures are reached near the seeds during burning (Figure 3A,B). Since the follicles open after a few seconds when directly exposed to a flame, the separator must also have an important function as an insulator, besides its hydration-driven ability to gradually pull the seeds out of open follicles, as suggested by Cowling and Lamont (1985). This is evident from the smooth and gradual increase of *T_seed_* in all species (Figure 2); that is, the lack of a sudden increase in temperature upon follicle opening. Among the three species, *B. serrata* shows the lowest maximum temperatures near the seeds (*T_seed,max_* ≈75°C), followed by *B. prionotes* and *B. candolleana* (*T_seed,max_* ≈90°C and 95°C, respectively) for similar surface temperatures in the range of 400–500°C (Figure 3B). These interior temperatures should not be lethal to the seeds inside [data for *B. prionotes* provided by Tangney et al. (2018)], especially if they have a relatively low moisture content that may be expected of mature seeds stored inside woody follicles (Tangney et al., 2018).

In *B. prionotes*, the fast transfer of heat through the valves is not problematic, because the separator provides additional insulation before heat propagates further toward the seeds. In *B. candolleana*, however, the arrangement of the follicles is different, and the valves form the main barrier between the seeds and the exterior. Due to their thick valves, heat can only propagate slowly, which might also lead to a delayed opening in comparison with follicles of *B. prionotes*. In *B. serrata*, heat transfer rates through the valves are also rather low, because of the relatively thick valves. At the same time, due to the embedment of the follicles, heat needs to pass the separator as well. However, owing to the larger distance *s* in *B. serrata*, this process takes more time than in *B. prionotes*. These two effects seem to add up in *B. serrata* follicles and result in the slowest heat transfer from the exterior toward the seeds among all three investigated species, as reflected by the experimental burns. The simple 2D FE-simulations, although predicting faster heating rates than those measured experimentally, give qualitative support to the differences between *B. prionotes* and *B. serrata*. The lower heating rate simulated in *B. candolleana* may arise due to the role of the external 3D geometry and needs to be investigated in more detail in the future. We link the low rates of heat transfer in *B. serrata* to the particular arrangement of follicles, in which thick valves are combined with an intermediate embedment.

For the interpretation of this result, we consider the presence or absence of persistent florets (meaning the retention of dried remnant flowers beyond the flowering time; strongly pronounced in *B. serrata*, Figure 1B), because the surface temperatures of follicles during crown fires might differ between the three investigated *Banksia* species. As Lamont and Cowling (1984) demonstrated, persistent floral remnants, such as present in *B. serrata*, and, in small amounts in *B. candolleana*, prolong the burning time substantially and increase surface temperatures as opposed to cones without floral remnants, such as in *B. prionotes*. Therefore, the presence of many persistent florets could explain why *B. serrata* follicles generally have a higher insulation capability – the follicles might not only face higher temperatures during fire due to the presence of highly flammable florets, but they might also experience fire for a longer time than *B. prionotes*, for example. Along the same lines, Judd (1993) claims that small Myrtaceae capsules have no special properties to insulate seeds, but are instead protected by short flame residence times promoted by the fine branches and architecture of the species.

The patterns of fire response vary amongst the three *Banksia* species; *B. serrata* and *B. candolleana* resprout (epicormically and lignotuberously, respectively) while *B. prionotes* is fire-killed (Collins et al., 2008). Some resprouting *Banksia* species are extremely long lived (“apparently immortal”; Drechsler et al., 1999), with *B. candolleana* estimated loosely to live for ∼1000 years (He et al., 2011). At the other extreme, *B. prionotes* lives no longer than the inter-fire period. Considering that *B. prionotes* is a fire killed species that regenerates exclusively from seeds, this species might benefit from investing only small amounts of biomass into producing an individual follicle, but then produces a large number of follicles per cone. Moreover, the cones are located higher in the canopy than those of the sympatric re-sprouting *B. candolleana*, which results in shorter exposure times to flames. Despite these extremes, beyond resprouting, *Banksia* show limited variation in functional traits that relate to fire: all are long-lived, generally surviving at least as long as the typical fire cycle; flowering is independent of fire; serotiny is a key trait for seed persistence, but varies within and between species, and; other than resistance to high temperatures and serotiny, with subsequent post-fire dispersal, seeds appear to have few fire-related adaptations. Our results show that whilst follicle size and geometry varies amongst the three species of *Banksia*, in all species the same function is provided – protection of seeds against the lethal temperatures during fire – and it is this seed protection function, regardless of the differences in follicle geometry that engenders it, that is central to the persistence of *Banksia* in fire prone environments.

During fire, the seeds are likely to benefit from chemical protection based on the prevalence of hydroxyl-rich condensed tannins in the valve tissue of all three *Banksia* species, especially in *B. serrata*. It is known that flames contain radicals, which can be responsible for ignition and radical-induced chain reactions during burning (Wilson and Fristrom, 1963), which are particularly harmful to the DNA, proteins, and cell membrane lipids of the seeds. Polyphenols, such as the condensed tannins in the valve tissue, can inhibit these free-radical reactions by forming highly stable quinones (Loudon and Parise, 2009). This reaction is promoted by the reductive O-H bonds that are found in the ring substituents of the condensed tannins. These hydroxyl groups react easily with radicals and form quinones, which are also efficient inhibitors of oxidation reactions, especially at low oxygen concentrations (Denisov and Denisova, 1999). Moreover, the network of condensed tannins might also contribute to maintain the integrity of the fibre bundles within the valve tissue (Figure 7A) at high temperatures, which is important to ensure the generation of mechanical forces for follicle opening (Wardrop, 1983; Huss et al., 2018).

The valve tissues of *B. serrata* and *B. candolleana* show a similar thermal conductivity as hardwoods, such as birch wood measured perpendicular to the grain, and with more precise measurement methods than in our study (Suleiman et al., 1999). In general, plants are capable of synthesising tissues with even lower thermal conductivities, such as the bark of the cork oak, also found in fire-prone regions, with *k* = 0.045 W/mK (Silva et al., 2005). However, with an average density of ϱ = 200 kg/m^3^ and a specific heat of *c* = 1900 J/kgK (Silva et al., 2005), we obtain almost the same thermal diffusivity for cork (α = 1.18E-07 m^2^/s) as in *B. prionotes* follicle valves (α = 1.19E-07 m^2^/s).

Since many canopy-stored seeds in Australia are able to withstand high temperatures outside of their cones (e.g., Hanley and Lamont, 2000; Tangney et al., 2018), there may be no requirement for even better thermal insulation. For example, seeds of *B. prionotes*, when dry, have a T50 lethal temperature of c. 140 °C when exposed to heat for 3 min (Tangney et al., 2018). Unfortunately, no data for longer exposure times (*t* > 3 min) or the corresponding lethal temperatures are available for seeds of the three tested *Banksia* species, which might be relevant, because the follicles need time to cool down. For seeds inside follicles of *B. serrata*, heating at *T* = 150°C for *t* = 10 min had no effect on seed viability (Bradstock, 1991), which indicates that their seeds might be able to tolerate the temperatures that we have measured experimentally in the range of *T_seed,max_* = 75°C. In different species of *Hakea*, a large proportion of the seeds is even able to survive direct exposure to *T* = 100°C for *t* = 10 min (Hanley and Lamont, 2000). In this context, it is important to remember that the follicles are multifunctional composites, in which thermal insulation is an important, but not the only requirement. One way to decrease heat diffusion would be to increase the tissue density, or the valve thickness. However, this might come along with a higher resource investment and other problematic changes for the plant, e.g., alternations in the opening mechanics and drying rates. Thus, the overall follicle properties should be interpreted as a finely balanced compromise between competing structural requirements for a variety of functions and environments.

## Materials and Methods

### Sample Collection and Preparation

Mature fruit cones of *B. serrata* were obtained from the Banksia Farm in Mt Barker, Western Australia, in September 2016. Mature cones of *B. prionotes* and *B. candolleana* were collected in the field in WA from the following locations: 31.46631°S, 115.66548°E and 31.43868°S, 115.66605°E (*B. prionotes*, May-June 2014) and 30.063759°S, 115.311367°E (*B. candolleana*, September 2016) and stored in cotton bags inside the lab until further processing. Individual follicles were then isolated from the cone with a band saw for each experiment.

### Experimental Burns

For the experimental burns, three follicles of each species were tested (originating from different cones in *B. candolleana*, and the same cone in *B. serrata* and *B. prionotes*). A small hole with a diameter of 1–2 mm was drilled into the follicles for inserting the thermocouple from the back, where the follicles are connected to the central axis of the rachis (Figure 2D). Afterward, we glued (loctite 454, Henkel) pieces of robinia wood onto the former rachis parts in order to protect the cables from the flames and to stabilise the samples during the experiment. The thermocouple (thermo element K406-484, TC Direct) near the seeds was inserted through the hole to a depth of 5 mm (*B. prionotes*) and 8 mm (*B. serrata* and *B. candolleana*), fixed in its position with a drop a of glue and covered with cotton wool to seal the hole from the back. The other thermocouple of the same kind was always placed on the upper follicle valve (∼0.8–1 cm away from the follicle tip for *B. serrata* and *B. candolleana*), where the thermo element was in direct contact with the valve surface, as illustrated in Figure 2E. For burning, a standard Bunsen burner flame, maintained by a butane-propane mixture of 80:20 (Camping Gaz), was manually rotated around the follicle for 180 s. After the experiment, the separator and the seeds were removed to double check whether the inner thermocouple (shown in Figure 2E) was placed centrally; this was usually indicated by scratches on the seed surface. During the experiment, the temperatures of the two thermocouples were recorded every second.

### 3D Imaging via X-Ray Microtomography

Individual follicles were scanned with an X-ray microtomography scanner (Easytom, RX-Solutions), equipped with a micro-focus tube (XRay150, RX-Solutions) and a flat panel detector (CsI scintillator). Scans were performed with a tube voltage of *U* = 60 kV, a tube current of *I* = 140 mA and an acquisition of *n* = 1440 filtered back projections in the continuous rotation mode (with reference images). Image stacks were reconstructed with a cone beam algorithm in the X-ACT software (RX-Solutions). For visualisation, 3D renderings and slice alignment for the longitudinal views were performed in Amira (Version 6.5, FEI).

### Thermal Conductivity Measurements

First, we calibrated the self-built set-up with two known standards (PVC with *k* = 0.24 W/mK, ϱ = 1.35 g/cm^3^ and PTFE with *k* = 0.15 W/mK, ϱ = 2.06 g/cm^3^) that were cut into rectangular pieces with a cross-sectional area of *A* = 9.3 mm × 10.7 mm, and a thickness of *x* = 3.35 mm and *x* = 6.0 mm. These standards were measured to determine the heat loss *P_loss_* of the set-up by inserting them into a holder made out of balsa wood, insulating five out of six surfaces of the sample. The uncovered surface was placed into the focus of an infrared (IR) heating spot (focused system, Optron) at a distance of 5 cm from the source. In front (heated surface) and behind the sample, we placed a thermocouple to record the surface temperatures (front thermocouple was covered with a thin strip of balsa wood to avoid direct exposure). In order to prevent air flow, a plastic box was attached around the set-up, which was, however, lacking thermal regulation. The power of the IR-spot was regulated via an external power supply unit (HCS-3202, Manson) set to a current of *I* = 2.0 A and a voltage of *U* = 1.3 V, which resulted in an average temperature in the focal spot of *T* = 43–44°C. For these settings, the optical power in the focal spot was *P_source_* = 0.206 W, as revealed by an optical power metre (PM206, ThorLABS), equipped with a broadband IR sensor sensitive to wavelengths in the range of λ = 185–20,000 nm (S310C, ThorLABS). For each sample, the temperature difference between the two sides (Δ*T*) and the heat flow through the sample *P_sample_* were determined after *t* = 24 h of heating by averaging over the last recorded hour. Before measuring samples of *B. candolleana*, *B. serrata* and balsa wood, we first calculated *P_loss_* for PVC and PTFE according to Eqs 4 and 5.

(4) $$P_{\mathrm{sample}}=k\Delta T\frac{A}{x}$$

(5) $$P_{\mathrm{loss}}=P_{\mathrm{source}}-P_{\mathrm{sample}}$$

As a consequence of insufficient insulation, *P_loss_* was relatively high (90.2% for 6.06 mm thick PVC and 85.3% for 3.13–3.35 mm thick PTFE and PVC). Due to the higher loss in thicker samples, with a thickness comparable to the follicle valves, we chose to use *P_loss_* = 90.2% for all measurements. Nonetheless, with this choice, we obtained acceptable results for balsa wood (⊥), with *k* = 0.07 W/mK, which lies inside the range of what has been reported in the literature (Nathan et al., 2014). For the measurements of follicle valves, we trimmed pieces of *B. serrata* and *B. candolleana* valves into similar dimensions as the references (only *A*, not their thickness) and measured three samples per species (originating from different follicles). Each sample contained all tissue layers (endo-, meso-, exocarp, as shown in Figure 5A) and was heated on its outer surface (cuticle, shown in Figure 5B). For slightly curved samples, the endocarp was flattened manually with a scalpel to ensure proper contact between the sample and the thermocouple in the back. Before sample mounting, the density was determined by normalising the sample weight to the volume (at a moisture content of ∼7.9%). After measuring all ‘native’ samples, the outer surface of the cubes was exposed to a flame for *t* = 30 s and then mounted back into the set-up to determine the thermal conductivity of samples with a burnt surface (using eq. 4 and 5).

### Model

Exemplary longitudinal 2D sections of all three species were traced and imported into Mathematica (Wolfram Mathematica, Version 11.3, RRID:SCR_014448). The sections were meshed using in-built meshing algorithms with a mesh size chosen such that the simulation results no longer vary with increasing refinement. A constant temperature was applied to the outer follicle surfaces giving the same initial temperature gradient as the experiments: Δ*T (B. prionotes)* = 414°C (Figure 2A1); Δ*T (B. serrata)* = 431°C (Figure 2B2); Δ*T (B. candolleana)* = 376°C (Figure 2, 2C1 and Table 1). A thermal diffusivity of α = 1.9 E-05 m^2^/s was used for air (Engineering ToolBox, (2018). Air – Thermal Diffusivity ^1^). As a first approximation, the other boundaries were assumed to have no heat flux. The heat equation was then solved using the in-built finite element solver on the experimental meshes.

### FT-IR Spectroscopy

Transverse slices of follicle valves with 6 μm thickness were cut with a rotary microtome (RM2255, Leica) and fixed onto plastic support strips for the measurements with a Fourier-transform IR microscope (Hyperion, 2000, Bruker) in combination with a mercury cadmium telluride detector. For each measurement area within the parenchymatic tissue, absorbance spectra in the range of 4000–600 cm^−1^, with a spectral resolution of 2 cm^−1^, were recorded using the software Opus 6.5 (Bruker). Each spectrum represents an average of 32 scans. Imaging of sample tissues was performed with a digital microscope (VHX-S550E, Keyence), equipped with a VH-Z100UR lens (Keyence), and used for mapping of the measurement areas. Based on these image maps, the distance to the valve surface was determined for each measurement area. For the burnt samples, this procedure was applied after exposing one valve to the flame of a Bunsen burner for *t* = 30 s and *t* = 60 s, respectively. The spectra for the charred tissue were recorded from a section heated in between two glass slides with a flame until its colour turned black. All spectra were analysed in Opus (Version 6.5, Bruker) and their baseline corrected using one iteration step in the in-built linear rubber band method of the software.

## Data Availability

All experimental raw data is stored at the MPIKG and all simulation raw data is stored at the University of Salzburg. The raw data supporting the conclusions of this manuscript will be made available by the authors, without undue reservation, to any qualified researcher upon request.

## Author Contributions

PF, ME, DM, and JH conceptualised and designed the study. JH performed the experimental work and wrote the first manuscript draft. JD and ME undertook the theoretical part of the study. JH, PF, and ME analysed the experimental data. DM and BM contributed with support during sampling and critical discussions. All authors contributed to data interpretation, manuscript revision and read and approved the submitted version.

## Conflict of Interest Statement

The authors declare that the research was conducted in the absence of any commercial or financial relationships that could be construed as a potential conflict of interest.

## Footnotes

1 https://www.engineeringtoolbox.com/air-thermal-diffusivity-d_2011.html [accessed November 29, 2018].
